# CXCL4 and CXCL4L1 Differentially Affect Monocyte Survival and Dendritic Cell Differentiation and Phagocytosis

**DOI:** 10.1371/journal.pone.0166006

**Published:** 2016-11-09

**Authors:** Mieke Gouwy, Pieter Ruytinx, Egle Radice, Federico Claudi, Katrien Van Raemdonck, Raffaella Bonecchi, Massimo Locati, Sofie Struyf

**Affiliations:** 1 KU Leuven, University of Leuven, Department of Microbiology and Immunology, Rega Institute for Medical Research, Laboratory of Molecular Immunology, Leuven, Belgium; 2 Humanitas Clinical and Research Center, Rozzano, Italy; University of Bergen, NORWAY

## Abstract

Upon inflammation, circulating monocytes leave the bloodstream and migrate into the tissues, where they differentiate after exposure to various growth factors, cytokines or infectious agents. The best defined macrophage polarization types are M1 and M2. However, the platelet-derived CXC chemokine CXCL4 induces the polarization of macrophages into a unique phenotype. In this study, we compared the effect of CXCL4 and its variant CXCL4L1 on the differentiation of monocytes into macrophages and into immature monocyte-derived dendritic cells (iMDDC). Differently to M-CSF and CXCL4, CXCL4L1 is not a survival factor for monocytes. Moreover, the expression of the chemokine receptors CCR2, CCR5 and CXCR3 was significantly higher on CXCL4L1-treated monocytes compared to M-CSF- and CXCL4-stimulated monocytes. IL-1 receptor antagonist (*IL-1RN*) expression was upregulated by CXCL4 and downregulated by CXCL4L1, respectively, whereas both chemokines reduced the expression of the mannose receptor (*MRC*). Furthermore, through activation of CXCR3, CXCL4L1-stimulated monocytes released significantly higher amounts of CCL2 and CXCL8 compared to CXCL4-treated monocytes, indicating more pronounced inflammatory traits for CXCL4L1. In contrast, in CXCL4L1-treated monocytes, the production of CCL22 was lower. Compared to iMDDC generated in the presence of CXCL4L1, CXCL4-treated iMDDC showed an enhanced phagocytic capacity and downregulation of expression of certain surface markers (e.g. CD1a) and specific enzymes (e.g. *MMP-9* and *MMP-12*). CXCL4 and CXCL4L1 did not affect the chemokine receptor expression on iMDDC and cytokine production (CCL2, CCL18, CCL22, CXCL8, IL-10) by CXCL4- or CXCL4L1-differentiated iMDDC was similar. We can conclude that both CXCL4 and CXCL4L1 exert a direct effect on monocytes and iMDDC. However, the resulting phenotypes are different, which suggests a unique role for the two CXCL4 variants in physiology and/or pathology.

## Introduction

Monocytes and macrophages are critically important in the regulation of innate and adaptive immune responses by generation of inflammatory mediators, antigen presentation, phagocytosis and killing of microorganisms. In contrast to resident tissue macrophages, which are long-living cells, monocytes in the circulation normally live for just a few days before undergoing apoptosis, a process finely tuned by caspases [1,2]. In the tumor microenvironment and in chronic inflammatory diseases, inhibition of the apoptotic program promotes monocyte survival, contributing to the accumulation of macrophages and the persistence of an inflammatory milieu. *In vitro*, the macrophage colony-stimulating factor (M-CSF) inhibits apoptotic processes and promotes the differentiation of monocytes into resting macrophages [3,4]. In response to different microenvironmental cues macrophages can adopt a polarized phenotype, exemplified in the M1/M2 paradigm [5]. Classical macrophages, also alluded to as M1 macrophages for their ability to assist in a T helper-1 (Th1) immune response, are induced by interferon-γ (IFN-γ) in combination with pathogen-derived signals (such as lipopolysaccharide; LPS) or pro-inflammatory cytokines (interleukin-1β; IL-1β and/or tumor necrosis factor-α; TNF-α), and are characterized by the production of significant amounts of inflammatory cytokines (IL-1β, IL-6, IL-12, IL-23, TNF-α) and reactive oxygen and nitrogen species [5,6]. On the contrary, alternatively activated or M2 macrophages evolve upon interaction with IL-4/IL-13, support Th2 responses [6,7] and promote fibrosis and tissue remodeling, via the production of transforming growth factor β (TGF-β) and matrix metalloproteinases (MMPs), respectively. M2 macrophages are characterized by high levels of scavenger-, mannose- and galactose-type receptors, reduced production of pro-inflammatory mediators, and contribute to angiogenesis by secreting vascular endothelial growth factor (VEGF) [5,7,8]. *In vivo* evidence has shown that in many inflammation-associated diseases, including cancer, infectious and allergic conditions, macrophages behave as plastic cells modifying in time and space their expression and transcription profile along a continuous spectrum, having M1 and M2 macrophage phenotypes as extremes [9,10].

During acute vascular injury or chronic disease, activated platelets have been recognized to play an important role in the activation of monocytes, e.g. by the release of pro-inflammatory cytokines, such as IL-1, or chemokines, such as RANTES/CCL5, platelet factor-4 (PF-4)/CXCL4 and its variant CXCL4L1 [11,12]. CXCL4 forms heterodimers with classical growth factors and other chemokines, binds with high affinity to glycosaminoglycans [13,14], and has been shown to act via the CXCR3 splice variants, CXCR3A and CXCR3B, on T lymphocytes and microvascular endothelial cells, respectively [15–17]. CXCL4 also protects monocytes from spontaneous apoptosis and induces their differentiation into a distinct macrophage subtype, alluded to as M4 macrophages [18–20]. CXCL4-induced macrophages have a unique transcriptome distinct from both M1 and M2 macrophages, characterized by low expression of HLA-DR and lack of CD163 expression [18–21]. Of note, CXCL4-dependent loss of CD163 expression is irreversible, suggesting that CXCL4-induced macrophages represent a final stage of cell differentiation, in contrast to M1 or M2 polarized macrophages [20]. The impact of CXCL4 on the regulation of cell growth has been shown in many other reports, demonstrating the inhibition of tumor growth and angiogenesis by CXCL4 [22–25]. Moreover, the CXCL4 variant CXCL4L1, isolated from thrombin-stimulated platelets and differing from authentic CXCL4 in three carboxy-terminally located amino acids, was found to be more potent than CXCL4 in inhibiting angiogenesis and tumor growth [26,27].

In this study, the effects of CXCL4 and its variant CXCL4L1 were investigated on the differentiation of monocytes into macrophages and immature monocyte-derived dendritic cells (iMDDC), by comparing the expression of several markers and chemokine/cytokine production. Finally, phagocytosis of *S*. *aureus* by CXCL4- or CXCL4L1-treated iMDDC was evaluated.

## Materials and Methods

### Reagents

Human recombinant macrophage colony-stimulating factor (M-CSF), IL-4, granulocyte macrophage colony-stimulating factor (GM-CSF) and CXCL4 were purchased from Peprotech (Rocky Hill, NJ, USA). Natural human CXCL4 was isolated from stimulated platelets as previously described [26]. Recombinant human CXCL4L1 was produced in insect cells and purified by a 4-step chromatographic procedure [17]. The ATPlite assay kit was obtained from Perkin Elmer (Boston, MA, USA). pHrodo red *S*. *aureus* bioparticles were obtained from Molecular Probes (Eugene, OR, USA). The CXCR3 antagonist AMG487 was purchased from R&D Systems (Minneapolis, MN).

### Cells

**Fig 1:**
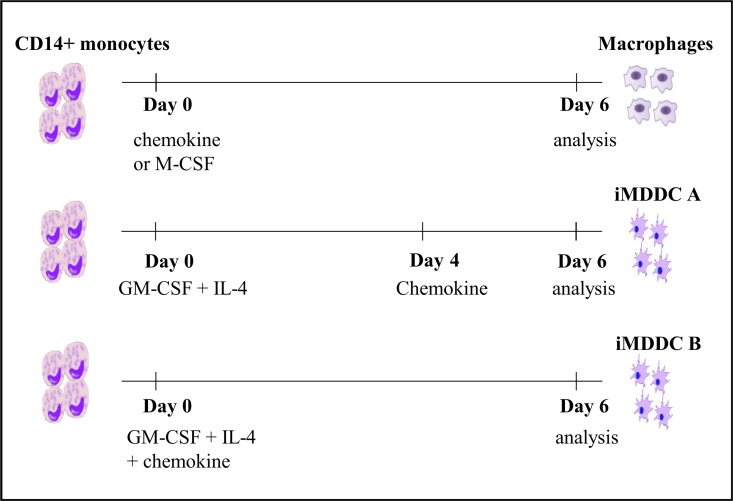
Treatment schedules of CD14^+^ monocytes to generate macrophages or immature dendritic cells. CD14^+^ monocytes were cultured in the presence of M-CSF (30 ng/ml), CXCL4 (1 and 10 μg/ml) or CXCL4L1 (1 and 10 μg/ml) to differentiate into macrophages. For the differentiation into dendritic cells, CD14^+^ monocytes were cultured in the presence of GM-CSF (50 ng/ml) and IL-4 (20 ng/ml). On day 4 (iMDDC A) or day 0 (iMDDC B), different concentrations of CXCL4 (1 and 10 μg/ml) or CXCL4L1 (0.1, 1 and 10 μg/ml) were added.

Human CD14^+^ monocytes were isolated from buffy coats derived from healthy donors (Blood Transfusion Center, Mechelen, Belgium) as described [28]. To induce the differentiation of monocytes into macrophages, CD14^+^ monocytes were suspended at a concentration of 2x10^6^ cells/ml in RPMI1640 medium (Lonza, Verviers, Belgium) supplemented with 10% fetal calf serum (FCS; Hyclone, Cramlington, UK) and gentamycin (50 μg/ml) and cultured in 6-well plates (2 ml/well). The stimuli (M-CSF, CXCL4 or CXCL4L1) were added on the first day of culture to obtain the different types of macrophages (Fig 1). Immature monocyte-derived dendritic cells (iMDDC) were generated by incubating purified human peripheral blood CD14^+^ monocytes in 6-well plates at 1x10^6^ cells/ml (2 ml/well) in RPMI1640 medium containing 10% FCS, 50 ng/ml GM-CSF and 20 ng/ml IL-4 for 6 days. On day 0 (iMDDC B) or day 4 (iMDDC A), 200 μl medium containing different concentrations of CXCL4 or CXCL4L1 was added to the MDDC cultures (Fig 1). On day 6 of culture, supernatants were collected to determine cytokine levels by ELISA. The macrophages and iMDDC were collected from the 6-well plates by pipetting up and down. Subsequently, phosphate buffered saline (PBS) was added to each well (1 ml/well) and the remaining cells were detached through incubation for 10 min at 4°C. Cells were centrifuged and resuspended in the corresponding buffer to perform RNA extraction, phagocytosis assays, and flow cytometry.

### Flow cytometry analysis

Expression of surface molecules and chemokine receptors on macrophages, iMDDC A or iMDDC B after 6 days of culture was analyzed by flow cytometry. Cells were washed in PBS and suspended in Zombie Aqua viability dye (1 μl/1 x 10^6^ cells; BioLegend, San Diego, CA, USA) for 15 min to exclude dead cells from the analysis. Afterwards, cells were washed and incubated for 10 min at 4°C with FACS buffer (PBS + 2% FCS) to block the Fc receptors and subsequently stained (30 min on ice) with the following monoclonal anti-human antibodies: allophycocyanin (APC) mouse CD1a (clone HI149), APC mouse CD14 (clone 63D3) and phycoerythrin (PE) mouse CXCL16 (clone 22-19-12) obtained from Biolegend, PE mouse CD163 (clone GHI/61), fluorescein (FITC) mouse HLA-DR (clone LN3) purchased from eBioscience (San Diego, CA, USA), PE mouse CD80 (clone L307.4), PE mouse CD86 (clone 2331), APC mouse CD11b (clone ICRF44), BV421 mouse CXCR3 (clone 1C6; this antibody does not discriminate between CXCR3A and CXCR3B), BV421 mouse CCR5 (clone 2D7), BV421 mouse CCR2 (clone 48607), Alexa Fluor 647 mouse CCR1 (clone 53504), PE mouse MSR1/CD204 (clone U23-56), APC mouse CD36 (clone CD38) and FITC mouse CD209/DC-SIGN (clone DCN46) from BD Biosciences (Heidelberg, Germany). Thereafter, cells were washed twice and fixed with 0.4% formaldehyde in FACS buffer. Acquisition was performed using an LSRFortessa X-20 cell analyzer (BD Biosciences) and data were analyzed using FlowJo software (Tree Star, Ashland, OR, USA).

### Gene expression studies

Relative changes in gene expression were evaluated by quantitative polymerase chain reactions (qPCR). Total RNA extraction was performed with the RNeasy Mini Kit (Qiagen, Venlo, The Netherlands) according to the manufacturer’s protocol. Afterwards, RNA quantification was achieved using the Nanodrop2000 (Thermo Scientific, Waltham, MA, USA) and for each sample the same amount of total RNA was reverse transcribed into cDNA using the High Capacity cDNA Reverse Transcription Kit (Applied Biosystems, Foster City, CA). Relative changes in gene expression were evaluated by qPCR using the TaqMan Fast Universal PCR master mix (Applied Biosystems) and commercially available primers and probes (Table 1). Sample mixes were loaded on a 96-well MicroAmp plate (Applied Biosystems) and were analyzed on the 7500 Fast Real-time PCR system. Obtained Ct values were processed following the 2^-ΔΔCt^ method, with 18S ribosomal 1 RNA (*RN18S1*) serving as housekeeping gene [29].

**Table 1:** Primer and probe sequences for evaluated genes.

Gene	Primers (5'→3')	Probe (5'→3')
*RN18S1*	ATCGCTCCACCAACTAAGAAC	/5HEX/ACCACCCAC/ZEN/GGAATCGAGAAAGAG/3IABkFQ/
	ACGGACAGGATTGACAGATTG	
*CCL2*	CCTCTGCACTGAGATCTTCC	/56-FAM/ATAGCAGCC/ZEN/ACCTTCATTCCCCAA/3IABkFQ/
	GCCTCCAGCATGAAAGTCT	
*VEGFA*	TGAACTTCACCACTTCGTGAT	/56-FAM/TGCTCTACC/ZEN/TCCACCATGCCAAG/3IABkFQ/
	CCATGAACTTTCTGCTGTCTTG	
*NOS2*	GCAGCTCAGCCTGTACT	/56-FAM/TATTCAGCT/ZEN/GTGCCTTCAACCCCA/3IABkFQ/
	CACCATCCTCTTTGCGACA	
*HMOX1*	AGAATCTTGCACTTTGTTGCTG	/5HEX/AGCTGCTGA/ZEN/CCCATGACACCAAG/3IABkFQ/
	CGTTCCTGCTCAACATCCA	
*MMP-2*	CAGACTTTGGTTCTCCAGCTT	/56-FAM/CACCCTTGA/ZEN/AGAAGTAGCTGTGACCG/3IABkFQ/
	ATCGAGATGCCTGGAATG	
*MMP-8*	AGCGAGCCCCAAAGAATG	/5HEX/TGTAATTTG/ZEN/CGGAGGTGTTGGTCCA/3IABkFQ/
	TCCTTGCTCATGCCTTTCAG	
*MMP-9*	CGTCGAAATGGGCGTCT	/56-FAM/CCAGGAGGA/ZEN/AAGGCGTGTGC/3IABkFQ/
	ACATCGTCATCCAGTTTGGTG	
*MMP-12*	AATCTCGTGAACAGCAGTGAG	/5HEX/AAGTTTGTG/ZEN/CCTCCTGAATGTGTAGTCC/3IABkFQ/
	CCTGGATCTGGCATTGGAG	
*CXCR3A*	ACCCAGCAGCCAGAGCACC	/56-FAM/TGAGTGACC/ZEN/ACCAAGTGCTAAATGACGC/3IABkFQ/
	TCATAGGAAGAGCTGAAGTTCTCCA	
*CXCR3B*	TGCCAGGCCTTTACACAGC	/5HEX/CCCGTTCCC/ZEN/GCCCTCACAGG/3IABkFQ/
	TCGGCGTCATTTAGCACTTG	
*MRC1*	TCCATCTTCCTTGTGTCAGC	/56-FAM/TTCATGAGT/ZEN/AGGTTTAGCATCAATAATTTTTGGTCTTT/3IABkFQ/
	GGTTTTGGAGTAATATTCACTGTTCT	
*IL1RN*	TTGTCCTGCTTTCTGTTCTCG	/56-FAM/TCAGTGATG/ZEN/TTAACTGCCTCCAGCTG/3IABkFQ/
	CTGTCCTGTGTCAAGTCTGG	

All primer/probe sets were supplied as PrimeTime qPCR assays by Integrated DNA Technologies (IDT).

### ELISA

Cytokine levels (e.g. CCL22/MDC, CCL18/PARC, IL-10, TNF-α, IL-1β) in cell culture supernatants (sampled 6 days after culture initiation) of iMDDC and macrophages were determined using ELISA according to instructions from the manufacturer (R&D systems). The CXCL8/IL-8-, CCL3/MIP-1α- and CCL2/MCP-1-specific ELISAs were developed in our laboratory [30].

### Phagocytosis assay

Immature MDDC (1.5x10^6^ cells/ml, 100 μl/sample) were incubated for 30 min at 37°C with 33 μl/sample of pHrodo red *S*. *aureus* bioparticles, previously resuspended in Live Cell Imaging solution (Molecular Probes). Cells were then washed in FACS buffer, fixed in FACS buffer containing 0.4% formaldehyde, and the pHrodo fluorescence in iMDDC was analyzed by flow cytometry. The phagocytic activity was quantified by first subtracting the background fluorescence (determined by incubating cells with bioparticles at 4°C) and subsequently, the net fluorescence for CXCL4- and CXCL4L1-treated iMDDC was divided by the net fluorescence of control iMDDC (differentiated with GM-CSF/IL-4 without addition of chemokine).

### Statistical analysis

Data were first analyzed by the non-parametric Kruskal-Wallis test (Statistica 12.0) for comparison of multiple (n>2) groups before performing pairwise comparisons. The Mann-Whitney U test and Sign test were used to compare data from two unpaired or paired groups, respectively.

## Results

### CXCL4, but not CXCL4L1, promotes macrophage survival

**Fig 2:**
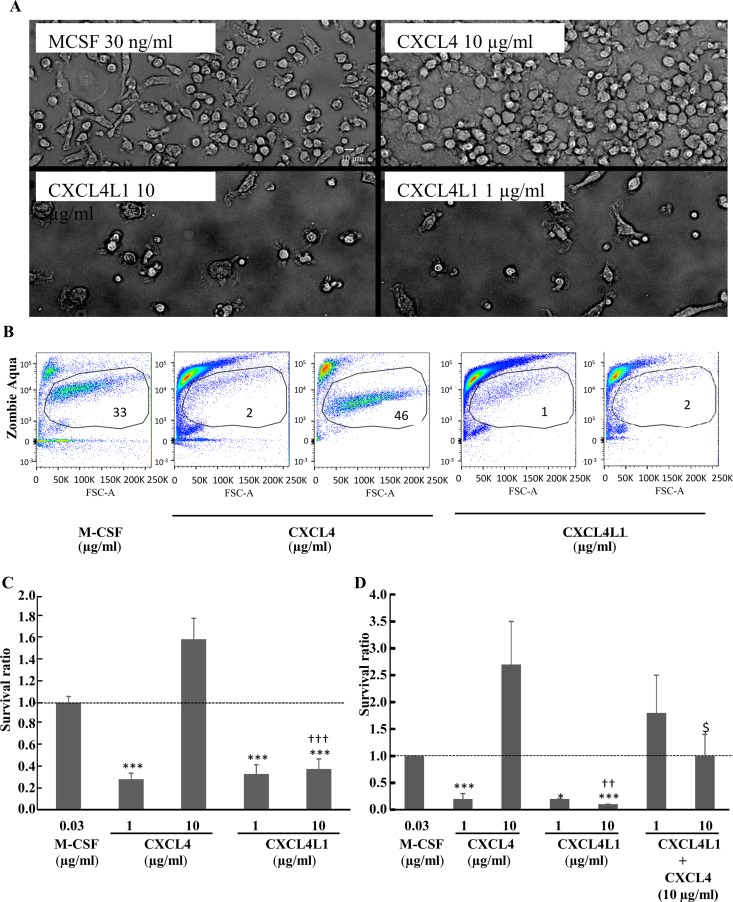
In contrast to CXCL4L1, CXCL4 induces morphological changes in monocyte-derived macrophages and promotes their survival. CD14^+^ monocytes were cultured in the presence of M-CSF (30 ng/ml), natural CXCL4 (1 and 10 μg/ml), recombinant CXCL4L1 (1 and 10 μg/ml) (panel A-C) or a combination of CXCL4 (10 μg/ml) and CXCL4L1 (1 and 10 μg/ml) (panel D). Panel A reports bright field pictures illustrating the morphology of monocyte-derived macrophages after 6 days of culture (20x magnification; scale bar 10μm). Panel B: After 6 days of culture cells were stained with the viability dye Zombie Aqua before flow cytometry analysis. Dot plots (forward scatter versus fluorescence) from one representative experiment out of 8 are shown. The gate circles the living cells. Numbers indicate the % of viable cells in the sample. Panel C and D report quantification of monocyte-derived macrophages after 6 days of culture by the ATP lite assay. The obtained luminescence values were normalized relative to the luminescence value obtained after culture in the presence of M-CSF (dashed line). Results are shown as mean ± SEM from 6–9 independent experiments. ***p<0.001; Mann-Whitney U test (CXCL4 or CXCL4L1 versus M-CSF) - ^†††^p<0.001; Mann-Whitney U test (CXCL4 versus CXCL4L1 at identical concentrations)—^$^p<0.05; Sign test [CXCL4 (10 μg/ml) versus the combination of CXCL4 (10 μg/ml) plus CXCL4L1 (10 μg/ml)].

It has been described that chemokines, in particular the platelet-derived CXC chemokine CXCL4, are involved in polarization and survival of monocytes [19]. CXCL4 stimulates monocyte survival and induces polarization into macrophages with a unique transcriptome [19]. However, the effect of CXCL4L1 on monocyte differentiation has never been determined. Therefore, human CD14^+^ monocytes were cultured for 6 days in the presence of CXCL4L1 (1 and 10 μg/ml). CXCL4 (1 and 10 μg/ml) and M-CSF (30 ng/ml) were included as control macrophage survival factors (Fig 1). In contrast to M-CSF (30 ng/ml) and CXCL4 (10 μg/ml), CXCL4L1 (1 and 10 μg/ml) did not stimulate monocyte survival (Fig 2). Microscopic analysis demonstrated that, after 6 days in culture, monocytes stimulated with 30 ng/ml M-CSF or with 10 μg/ml CXCL4 showed a normal cell density and a morphology characteristic of macrophages, whereas CD14^+^ monocytes stimulated with CXCL4L1 (1 and 10 μg/ml) were far less numerous and their numbers were comparable to those of unstimulated monocytes (Fig 2A and data not shown). Flow cytometry with the Zombie Aqua dye showed a lower % of living cells when CD14^+^ monocytes were treated with 1 or 10 μg/ml CXCL4L1 (i.e. 6.8±4.4% and 9.5±3.1%, respectively, *p*<0.005) compared to 30 ng/ml M-CSF (i.e. 47.7±6.4%) or 10 μg/ml CXCL4 (i.e. 56.4±5.7%) (Fig 2B). When the amount of intracellular ATP, which is a good parameter for cell viability and metabolic activity and thus the cell number in cultures, was quantified using an ATP assay kit, the number of macrophages after stimulation with CXCL4L1 (1 and 10 μg/ml) or 1 μg/ml CXCL4 was significantly lower compared to cell numbers in cultures treated with 10 μg/ml CXCL4 or 30 ng/ml M-CSF (Fig 2C). Moreover, CXCL4L1 (10 μg/ml) statistically inhibited macrophage survival induced by CXCL4 (10 μg/ml) (Fig 2D).

### CXCL4- and CXCL4L1-treated monocytes express different levels of metalloproteinases and chemokine receptors

**Fig 3:**
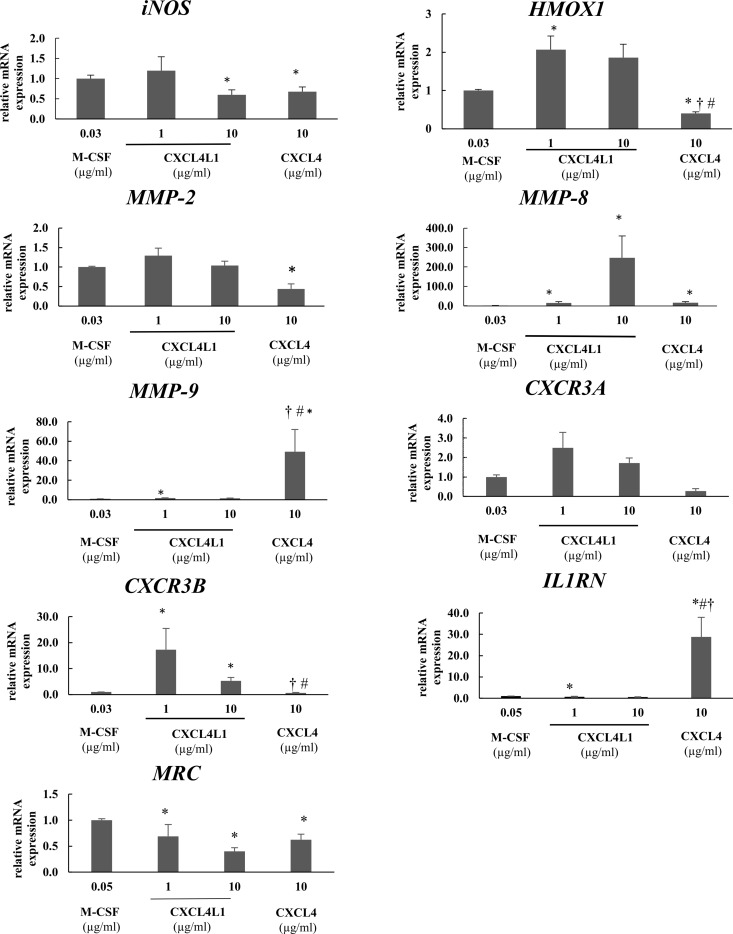
Effects of CXCL4 and CXCL4L1 on the matrix metalloproteinase and chemokine receptor gene expression in differentiated monocyte-derived macrophages. Relative expression of *iNOS*, *HMOX1*, *MMP-2*, *MMP-8*, *MMP-9*, *CXCR3A*, *CXCR3B*, *IL-1RN and MRC* was evaluated in monocyte-derived macrophages following 6 day-treatment with M-CSF- (30 ng/ml), CXCL4 (10 μg/ml) or CXCL4L1 (1 and 10 μg/ml). Results are shown as mean (± SEM) percentage expression relative to the expression in M-CSF-treated monocytes. *p<0.05, Mann-Whitney U test (CXCL4 or CXCL4L1 versus M-CSF); ^†^p<0.05, Mann-Whitney U test (CXCL4 versus CXCL4L1 at identical concentrations); ^#^p<0.05, Mann-Whitney U test (CXCL4 vs CXCL4L1 1 μg/ml).

In order to get insight in the transcriptome of CXCL4L1-stimulated monocytes, human CD14^+^ monocytes were exposed to M-CSF (30 ng/ml), CXCL4 (10 μg/ml) or CXCL4L1 (1 and 10 μg/ml) for 6 days. RNA was then extracted and the expression levels of several MMPs, the established M1/M2 markers (i.e. *iNOS* and *HMOX1*), the IL-1 receptor antagonist (i.e. *IL-1RN*), the IL-1 receptor, type II (*IL-1R2*), the mannose receptor (*MRC*), as well as both splice variants of the CXCR3 receptor, were assessed by qPCR (Fig 3). Among the MMPs, *MMP-9* showed significantly higher expression in CXCL4- and CXCL4L1 (1 μg/ml)-stimulated monocytes as compared to M-CSF-treated monocytes, with even significantly higher expression in CXCL4-treated monocytes compared to CXCL4L1-treated monocytes. Unlike the upregulation of *MMP-9*, we found a downregulation of *MMP-2* expression in CXCL4-induced macrophages, whereas *MMP-2* expression was slightly increased in CXCL4L1-treated monocytes. *MMP-12* expression was below detection limit (data not shown), but we found significantly increased expression of *MMP-8* in both CXCL4L1- and CXCL4-treated monocytes as compared to resting macrophages. We confirmed previous observations indicating that CXCL4 suppresses expression of the M2 marker heme oxygenase-1 (*HMOX1*) [20]. On the contrary, we found that *HMOX1* expression was significantly increased in CXCL4L1-treated monocytes. In contrast to previous reports [19], we also observed a significant downregulation of the M1 marker inducible nitric oxide synthase *iNOS* enzyme in CXCL4-differentiated monocytes, which was also evident in CXCL4L1-treated (10 μg/ml) monocytes.

The molecular identity of receptors involved in transducing CXCL4 effects in monocytes is still a matter of debate [15,25,31,32]. We investigated the expression levels of the candidate receptor *CXCR3* gene and found that, while CXCL4 had no effect, CXCL4L1 induced an increase in the expression levels of CXCR3A. Similar results were obtained for CXCR3B, the other splice variant of the CXCR3 receptor. Indeed, CXCL4-stimulated monocytes showed lower expression levels of the CXCR3B receptor compared to M-CSF, whereas CXCL4L1-treated monocytes showed significantly increased expression of the receptor CXCR3B. As shown in Fig 3, *IL-1RN* expression was increased in CXCL4-treated monocytes, whereas in CXCL4L1-treated monocytes the expression was slightly decreased compared to resting macrophages (Fig 3). Furthermore, *MRC* expression was significant downregulated in CXCL4- and CXCL4L1-treated monocytes as compared to resting macrophages. *IL-1R2* expression was below detection limit (data not shown).

### Monocytes cultured in the presence of CXCL4 or CXCL4L1 differ in their surface marker expression pattern

**Fig 4:**
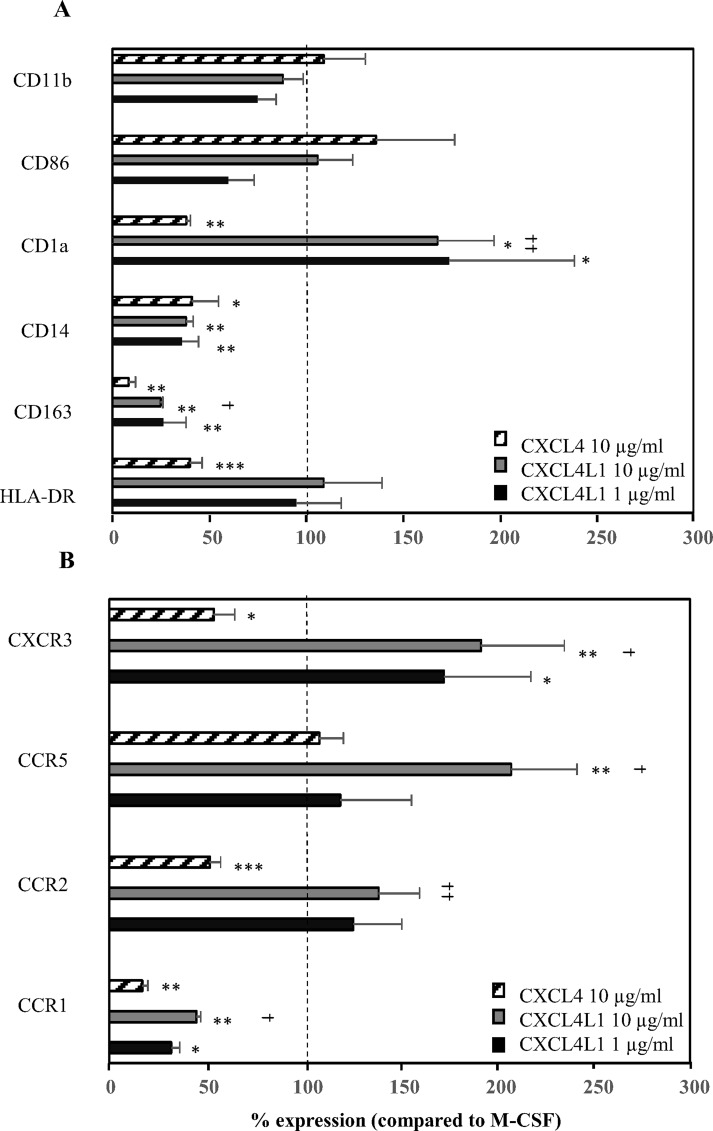
The expression of surface markers and chemokine receptors on macrophages after stimulation with M-CSF, CXCL4 or CXCL4L1. Purified human CD14^+^ monocytes were cultured for 6 days in the presence of recombinant M-CSF (30 ng/ml), natural CXCL4 (10 μg/ml) or recombinant CXCL4L1 (1 and 10 μg/ml). On day 6 expression of several surface markers (panel A) and chemokine receptors (panel B) was analyzed by flow cytometry. Results from 4 to 8 independent experiments were expressed as mean fluorescence intensity and are shown as mean (± SEM) percentages, normalized to M-CSF-treated monocytes (100%). *p<0.05, **p<0.01,***p<0.001, Mann-Whitney U test (CXCL4 or CXCL4L1 versus M-CSF); ^†^p<0.05,^††^p<0.01, Mann-Whitney U test (CXCL4 versus CXCL4L1 at identical concentrations).

To further characterize the phenotype of CXCL4- and CXCL4L1-stimulated monocytes, the expression of a panel of macrophage receptors was determined by FACS analysis. Similar to CXCL4-treated monocytes, CXCL4L1-stimulated monocytes have a statistically significant lower expression of CD14, the hemoglobin-haptoglobin scavenger receptor CD163, and the chemokine receptor CCR1 when compared to M-CSF-treated monocytes (Fig 4). The expression of the costimulatory molecule CD86, which is required for T cell activation by macrophages, was unaffected by monocyte exposure to CXCL4 and CXCL4L1, whereas the expression of CD1a was regulated in opposite ways in CXCL4- or CXCL4L1-treated monocytes (Fig 4A). The expression of the chemokine receptors CCR2, CCR5 and CXCR3 was significantly higher on CXCL4L1-treated monocytes as compared to CXCL4-treated monocytes (Fig 4B). Unlike its significant downregulation reported in CXCL4-induced macrophages [18], HLA-DR has expression levels in CXCL4L1-polarized macrophages comparable to M-CSF-treated cells. In summary, our data show that human macrophages generated in the presence of CXCL4 or CXCL4L1 have a distinct surface marker profile.

### Differential effect of CXCL4 and CXCL4L1 on chemokine production by monocyte-derived macrophages

**Fig 5:**
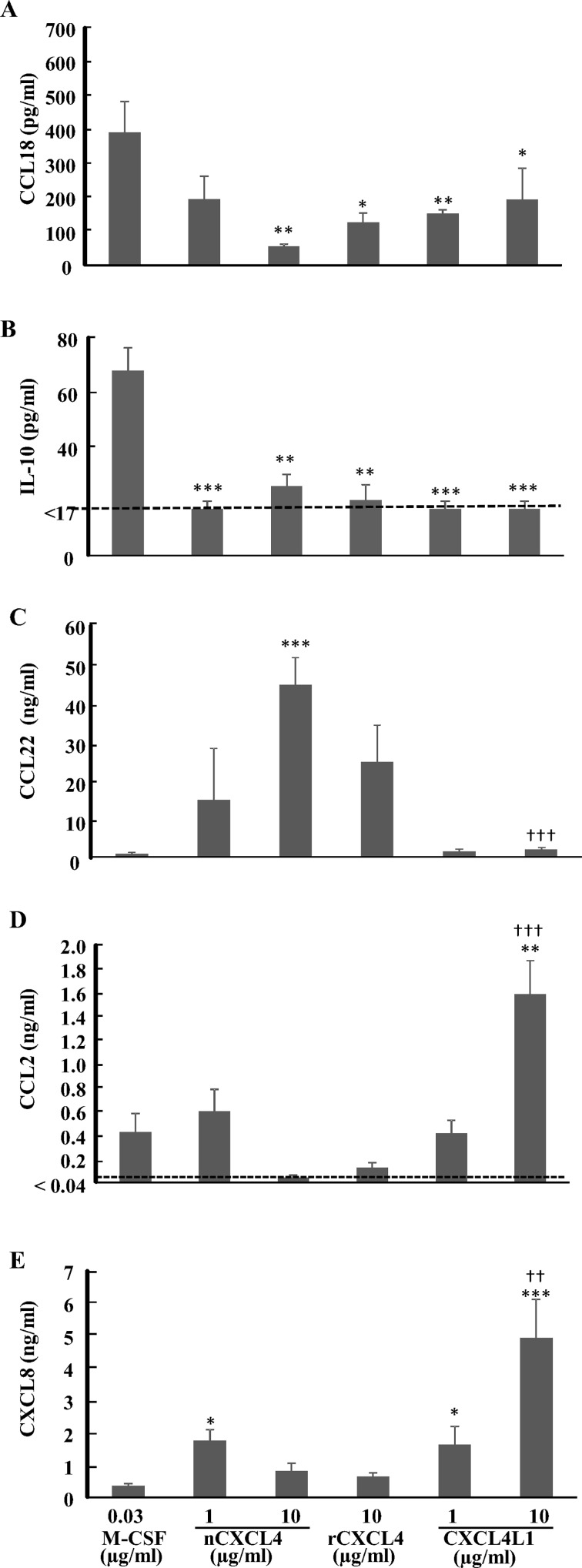
Cytokine and chemokine production by macrophages after stimulation with M-CSF, CXCL4 or CXCL4L1. CD14^+^ monocytes were differentiated in the presence of recombinant M-CSF (30 ng/ml), natural or recombinant CXCL4 (1–10 μg/ml) or recombinant CXCL4L1 (1 and 10 μg/ml). After 6 days of culture, cell supernatants were analyzed by ELISA to determine the amount of CCL18 (panel A), IL-10 (panel B), CCL22 (panel C), CCL2 (panel D) and CXCL8 (panel E). The measured cytokine concentrations were corrected for the number of viable cells in the cultures at the time of sampling. The results are shown as mean ± SEM and are pooled from 8 independent experiments. *p<0.05, **p<0.01,***p<0.001, Mann-Whitney U test (CXCL4 or CXCL4L1 versus M-CSF); ^††^p<0.01, ^†††^p<0.001, Mann-Whitney U test (CXCL4 versus CXCL4L1 at identical concentrations).

One of the consequences of polarized macrophage activation is the profoundly different set of cytokines and chemokines that is secreted [6,7]. The concentration of cytokines released in the supernatant by CD14^+^ monocytes cultured for 6 days in the presence of M-CSF (30 ng/ml), natural (1 and 10 μg/ml) or recombinant CXCL4 (10 μg/ml), or recombinant CXCL4L1 (1 and 10 μg/ml) was therefore measured by ELISA. Taking into account the number of living cells, no difference between CXCL4 and CXCL4L1 treatment was observed in the production of CCL18 and IL-10, both of which were released at levels significantly lower than after M-CSF treatment (Fig 5A and 5B). On the other hand, but in agreement with previous reports, CXCL4 enhanced the production of CCL22 [19]. Recombinant CXCL4 (10 μg/ml) and natural CXCL4 induced the release of comparable amounts of CCL22, excluding contamination of the natural CXCL4 preparation with other chemokines. In contrast, CXCL4L1-stimulated monocytes, secreted amounts of CCL22 comparable to M-CSF-treated monocytes, with only at 10 μg/ml a minor, yet statistically significant increase (Fig 5C). Interestingly, we measured significant production of inflammatory chemokines (CCL2 and CXCL8; Fig 5D and 5E, respectively) in monocytes stimulated with CXCL4L1 (10 μg/ml), whereas CXCL4 (10 μg/ml) did modulate inflammatory chemokine production in the same way as M-CSF. The production of TNF-α, IL-1β and CCL3 was below detection limit of the ELISA for all experimental groups (data not shown). We conclude that human macrophages generated in the presence of CXCL4 or CXCL4L1 are significantly different in their profile of secreted cytokines.

**Fig 6:**
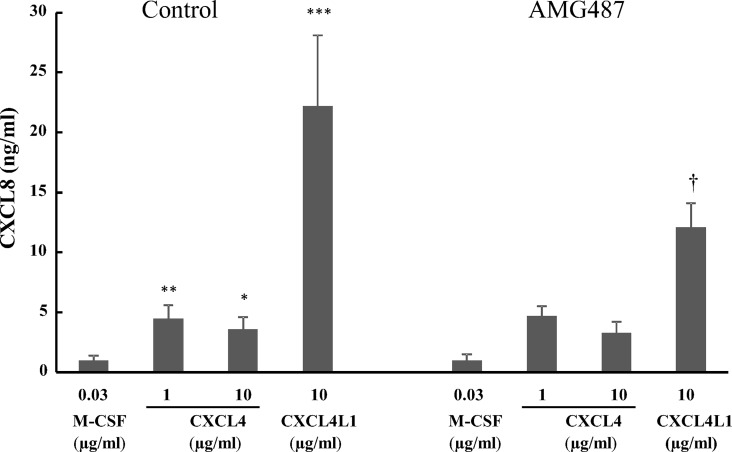
Chemokine production by macrophages after stimulation with CXCL4L1 is mediated by CXCR3. CD14^+^ monocytes were treated with control medium or AMG487 (100 nM) and differentiated in the presence of recombinant M-CSF (30 ng/ml), natural CXCL4 (1–10 μg/ml) or recombinant CXCL4L1 (10 μg/ml). After 6 days of culture, cell supernatants were analyzed by ELISA to determine the amount of CXCL8. The measured cytokine concentrations were corrected for the number of viable cells in the cultures at the time of sampling. The results are shown as mean ± SEM and are pooled from 8–10 independent experiments. *p<0.05, **p<0.01,***p<0.001, Mann-Whitney U test (CXCL4 or CXCL4L1 versus M-CSF); ^†^p<0.05, Sign test (inhibition by AMG487 of CXCL8 release).

To verify whether CXCR3 is involved in chemokine induction, we added the CXCR3 antagonist AMG487 during the full incubation period. This antagonist did not modulate the CXCL4-stimulated CXCL8 release. It must be noted, however, that CXCR3 expression in CXCL4-stimulated monocytes was significantly downregulated (Figs 3 and 4). In contrast, CXCL4L1 upregulated CXCR3 expression and CXCL8 production by CXCL4L1-treated monocytes was significantly inhibited in the presence of the CXCR3 antagonist AMG487 (Fig 6).

### Effects of CXCL4 and CXCL4L1 on the differentiation of monocytes into iMDDC

**Fig 7:**
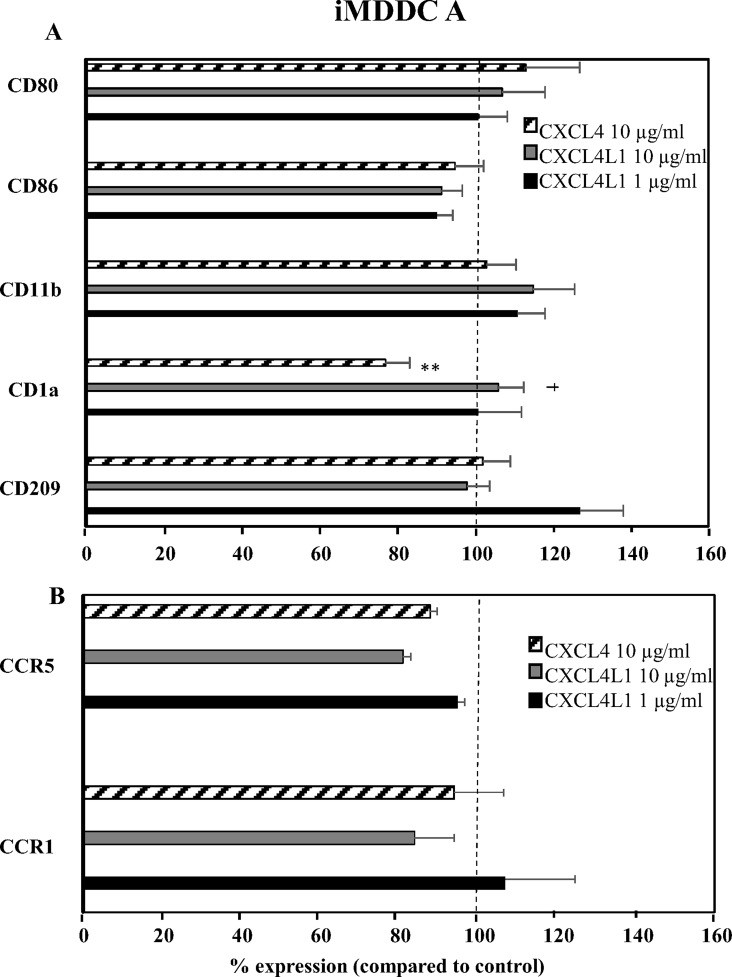
Modulation of the surface marker and chemokine receptor expression on immature MDDC by CXCL4 and CXCL4L1. Purified human CD14^+^ monocytes were cultured in the presence of 50 ng/ml GM-CSF and 20 ng/ml IL-4. On day 4, different concentrations of natural CXCL4 (10 μg/ml) or recombinant CXCL4L1 (1 and 10 μg/ml) were added to the cultures. On day 6 (i.e. 2 days after addition of chemokines), iMDDCs A were collected and the expression of surface markers (panel A) and chemokine receptors (panel B) was analyzed by flow cytometry. Results from 3 to 7 independent experiments were expressed as mean fluorescence intensity and are shown as mean (± SEM) percentages, normalized to iMDDC cultured in the absence (100%) of CXCL4 or CXCL4L1. **p<0.01, Mann-Whitney U test (CXCL4 versus untreated); ^†^p<0.05, Mann-Whitney U test (CXCL4 versus CXCL4L1 at identical concentrations).

**Fig 8:**
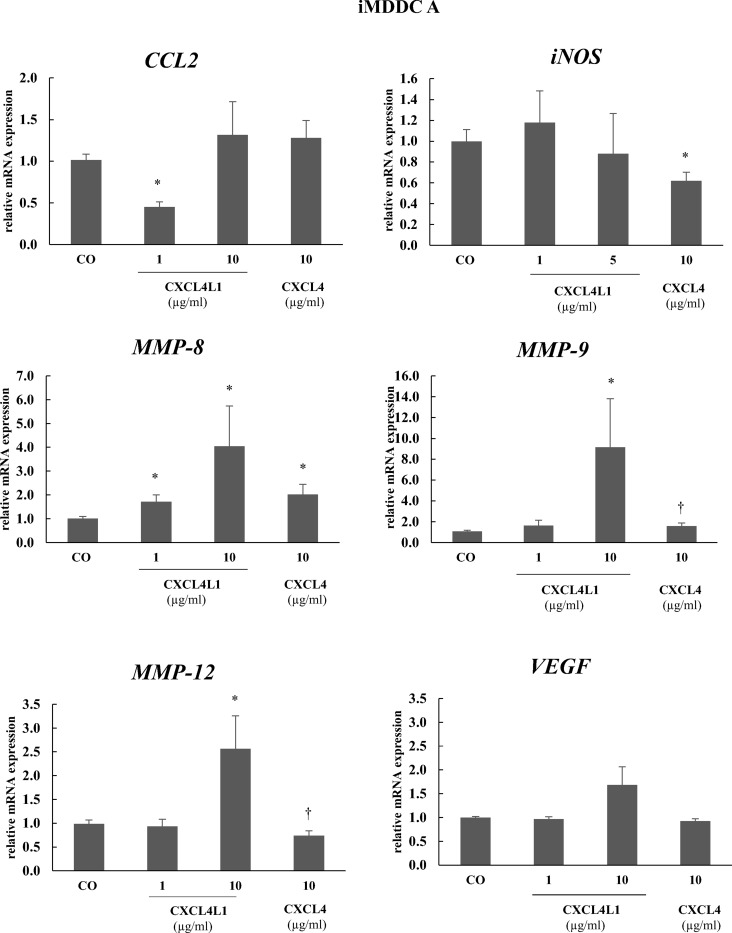
Effects of CXCL4 and CXCL4L1 on gene expression in differentiated immature MDDC. Relative expression of *CCL2*, *iNOS*, *MMP-8*, *MMP-9*, *MMP-12* and *VEGF* was evaluated in iMDDC A following treatment with GM-CSF (50 ng/ml) and IL-4 (20 ng/ml) in the absence (Co) or presence of CXCL4- (10 μg/ml) or CXCL4L1 (1 and 10 μg/ml). The results are shown as mean (± SEM) percentage expression relative to the expression on untreated iMDDC. *p<0.05, Mann-Whitney U test (CXCL4 or CXCL4L1 versus CO); ^†^p<0.05, Mann-Whitney U test (CXCL4 versus CXCL4L1 at identical concentrations).

Having observed differential effects of CXCL4 and CXCL4L1 on monocyte differentiation into macrophages, we investigated their effects on the differentiation of monocytes into iMDDC in response to GM-CSF and IL-4. Two different approaches were followed. In the first set of experiments, chemokines were added on day 4 (iMDDC A), whereas in the second set of experiments, CXCL4 or CXCL4L1 was added on day 0, together with GM-CSF and IL-4 (iMDDC B). After 6 days of culture, iMDDC A were analyzed by flow cytometry for the expression of different surface markers and chemokine receptors (Fig 7). We confirmed previous reports that iMDDC A express a significantly lower CD1a level in the presence of CXCL4 (10 μg/ml), compared to cells cultured in the absence of CXCL4 [31]. Neither CXCL4 nor CXCL4L1 did change the expression of DC-SIGN/CD209, CD11b, CD80, or CD86 on iMDDC A (Fig 7A). In contrast to CXCL4L1-stimulated monocytes, the expression of the chemokine receptors CCR1 and CCR5 was not affected by CXCL4L1 in iMDDC A (Fig 7B). We next determined the effects of CXCL4 and CXCL4L1 on gene expression in iMDDC A by qPCR (Fig 8), investigating *MMPs* and pro-inflammatory mediators *CCL2* and *iNOS*. Although at 1 μg/ml CXCL4L1 reduced CCL2 expression by iMDDC A, this observation was not confirmed at the protein level (*vide infra*). At 10 μg/ml, CXCL4, nor CXCL4L1 changed *CCL2* gene expression. However, we found a significant downregulation of *iNOS* expression by CXCL4 (10 μg/ml). Significant upregulation of *MMP-8* was observed by both CXCL4 (10 μg/ml) and CXCL4L1 (1 and 10 μg/ml) compared to control iMDDC A, whereas *MMP-9*, as well as *MMP-12* expression was significantly induced by CXCL4L1 (10 μg/ml) in iMDDC A, but remained unaffected by CXCL4 exposure.

### Effects of CXCL4 and CXCL4L1 on cytokine expression in immature MDDC

**Fig 9:**
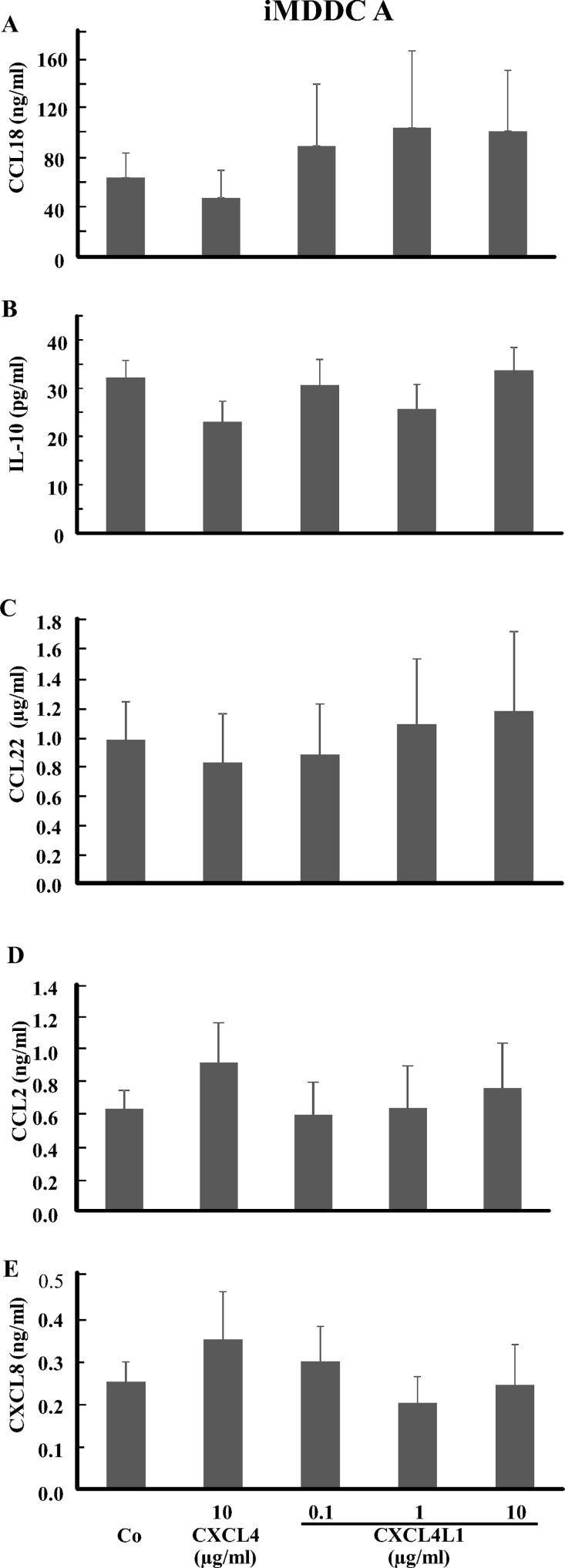
Cytokine and chemokine production by iMDDC A after stimulation with CXCL4 or CXCL4L1. Purified human CD14^+^ monocytes were cultured in the presence of 50 ng/ml GM-CSF and 20 ng/ml IL-4. On day 4, different concentrations of natural CXCL4 (10 μg/ml) or recombinant CXCL4L1 (0.1, 1 and 10 μg/ml) were added to the cultures. Cell supernatants were collected after 6 days of culture. The amount of CCL18 (panel A), IL-10 (panel B), CCL22 (panel C), CCL2 (panel D) and CXCL8 (panel E) produced was measured by sandwich ELISAs. The results (mean ± SEM) are derived from 5–7 independent experiments.

**Fig 10:**
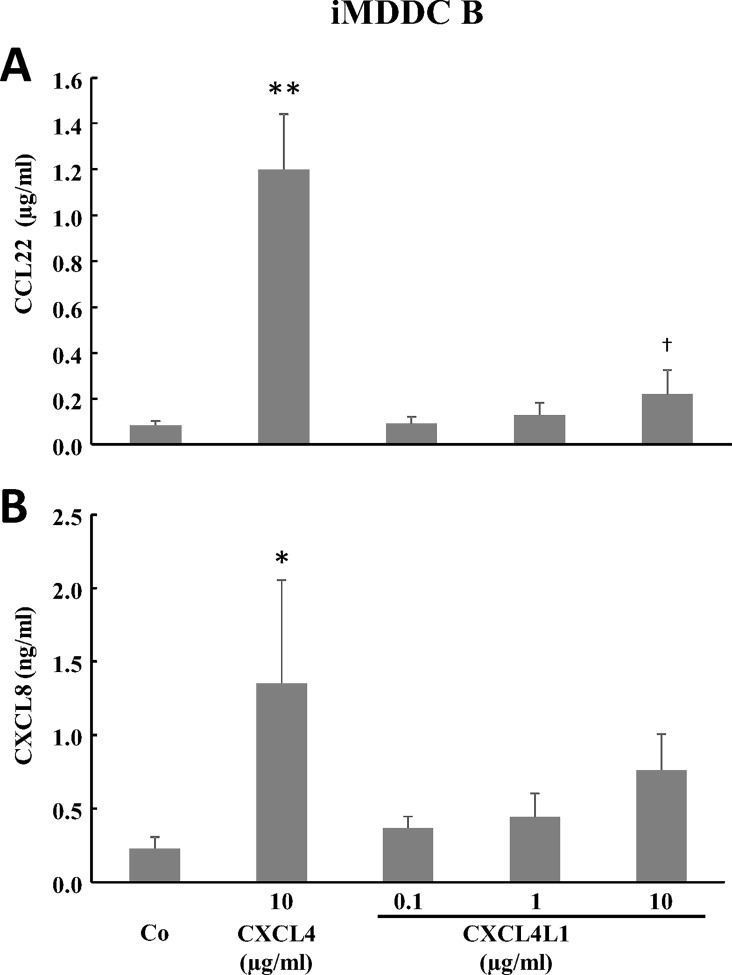
Chemokine production by iMDDC B after a 6 day-treatment with CXCL4 or CXCL4L1. Purified human CD14^+^ monocytes were cultured for 6 days in the presence of 50 ng/ml GM-CSF and 20 ng/ml IL-4. From the start of the culture different concentrations of natural CXCL4 (10 μg/ml) or recombinant CXCL4L1 (0.1, 1 and 10 μg/ml) were added (iMDDC B). Cell supernatants were collected after 6 days of culture. The amount of CCL22 (panel A) and CXCL8 (panel B) produced was measured by sandwich ELISAs. The results (mean ± SEM) are derived from 6 independent experiments. *p<0.05, **p<0.01, Mann-Whitney U test (CXCL4 or CXCL4L1 versus M-CSF); ^†^p<0.05, Mann-Whitney U test (CXCL4 versus CXCL4L1 at identical concentrations).

We next measured cytokine production by CXCL4-stimulated iMDDC A or CXCL4L1-stimulated iMDDC A. The levels of TNF-α, IL-1β and CCL3 in the supernatants of iMDDC A were below the ELISA detection limit (data not shown), whereas levels of CXCL8, CCL2, CCL18, CCL22, and IL-10 remained unchanged by either CXCL4 or CXCL4L1 exposure as compared to control iMDDCs (Fig 9). However, CXCL4-stimulated iMDDC B produced significantly more CCL22 and CXCL8 than control iMDDC. CXCL4L1 did not modulate CXCL8 or CCL22 production in either type of iMDDC (Fig 10A and 10B).

### Effects of CXCL4 and CXCL4L1 on S. aureus phagocytosis by immature MDDC

**Fig 11:**
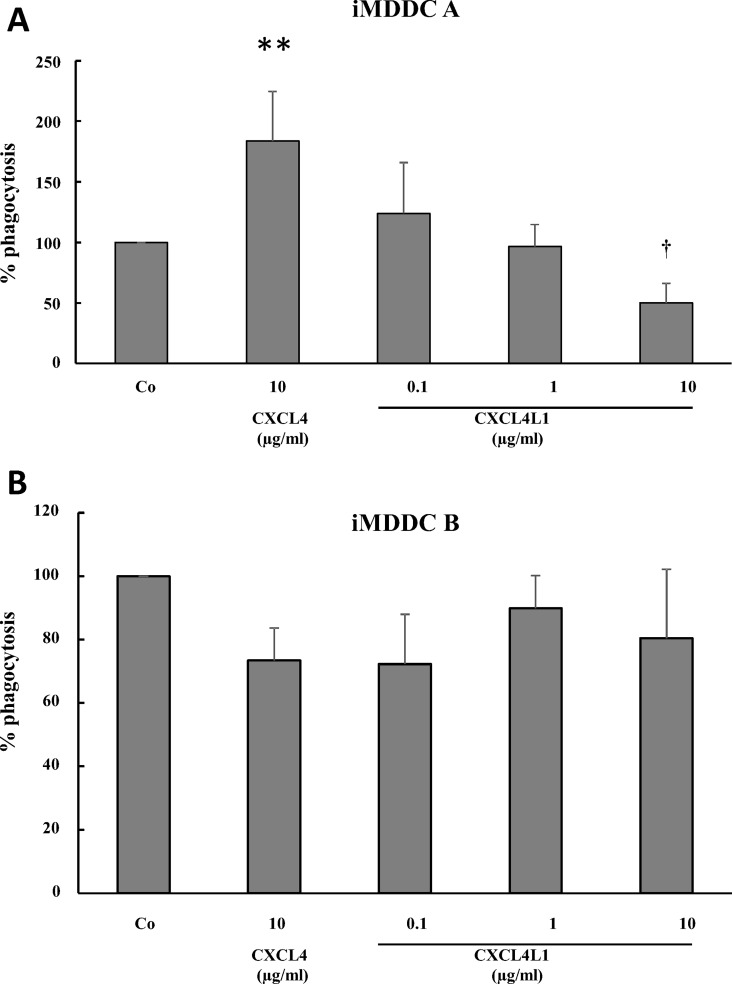
CXCL4- or CXCL4L1-stimulated phagocytosis of *S*. *aureus* by dendritic cells. CD14^+^ monocytes were cultured in the presence of 50 ng/ml GM-CSF and 20 ng/ml IL-4. On day 4 (panel A; iMDDC A) or day 0 (panel B;iMDDC B), different concentrations of natural CXCL4 (10 μg/ml) or recombinant CXCL4L1 (0.1, 1 and 10 μg/ml) were added. After 6 days of culture, DC were exposed to pHrodo-labeled *S*. *aureus* as described in *Methods*. The phagocytic capacity of CXCL4- and CXCL4L1-stimulated iMDDC was assessed by flow cytometry and is expressed relative to the net fluorescence of control iMDDC, differentiated with GM-CSF plus IL-4 without addition of chemokine (Co). Results of 3–4 independent experiments, each performed in duplicate are shown. *p<0.05; Mann-Whitney U test (CXCL4 versus M-CSF) - ^†^p<0.05; Mann-Whitney U test (CXCL4 versus CXCL4L1).

An important function of dendritic cells is to phagocytose pathogens and subsequently display pathogen-derived antigens to lymphocytes. To determine the phagocytic capacity of CXCL4-stimulated iMDDC A and CXCL4L1-stimulated iMDDC A, cells were exposed to pHrodo-labeled *S*. *aureus* beads. As shown in Fig 11A, iMDDC A cultured in the presence of CXCL4 (10 μg/ml) more actively phagocytosed the *S*. *aureus* beads (195.7±48%) compared to control iMDDC. In contrast, stimulation with CXCL4L1 (10 μg/ml) inhibited the phagocytic capacity of iMDDC A (35.5±11.4%). In contrast, CXCL4L1- and CXCL4-stimulated iMDDC B phagocytosed *S*. *aureus* beads as efficiently as control iMDDC (Fig 11B).

## Discussion

Inflammatory chemokines, e.g. CCL3, can mediate a short-term biological response of monocytes such as chemotaxis, without promoting their survival. Other chemokines, such as CXCL4, play, like M-CSF, a role in long-term regulatory processes by inhibiting apoptosis and inducing differentiation of monocytes into macrophages [18–20]. However, compared to M-CSF-induced macrophages, CXCL4 induces a macrophage phenotype, which shares similarities with both M1 and M2 macrophages [19]. In the present study, we report on the differential effect of CXCL4 and CXCL4L1 on monocyte survival and differentiation. In contrast to CXCL4-induced macrophages, CD14^+^ monocytes stimulated with CXCL4L1 were far less numerous, their numbers being comparable to those of unstimulated monocytes. Unlike CXCL4-stimulated monocytes [18], CXCL4L1-treated cells did not display significant downregulation of HLA-DR compared to M-CSF-induced macrophages, which suggests that their antigen-presenting capacity is preserved. On the other hand, a number of expression markers (e.g. CD11b, CD14, CD86, CD163) was not differentially expressed between CXCL4- and CXCL4L1-stimulated monocytes. CXCL4-induced macrophages have been reported to express considerably less surface CCR2 as compared to M-CSF-exposed cells [32]. We found, compared to CXCL4-induced macrophages and M-CSF-exposed cells, CXCL4L1-stimulated monocytes to display higher expression levels of the inflammatory chemokine receptors CCR2 and CCR5, suggesting that CXCL4L1 promotes a higher responsiveness to inflammatory CC chemokines, such as CCL2 and CCL3. In addition, the amount of CXCL8 and CCL2 (M1 marker) released was significantly higher in CXCL4L1-stimulated monocytes than in CXCL4-treated monocytes, whereas the concentration of CCL22 (M2 marker) was lower [6]. Though we could not confirm upregulated CCL18 synthesis, we reproduced data reported by Gleissner *et al*. [19], who detected higher production of CCL22 in CXCL4-stimulated monocytes. Based on Fig 6, we postulate that the CXCL4-induced production of CXCL8 is independent of CXCR3, whereas CXCR3 is implicated in the CXCL4L1-stimulated CXCL8 production by macrophages. Kasper and Petersen [13] already suggested that glycosaminoglycans mediate the CXCL4-induced monocyte survival. Expression of *iNOS* (M1 marker) in CXCL4L1-stimulated monocytes was decreased compared to M-CSF-treated monocytes, whereas the levels of *HMOX1* (M2 marker) expression upon CXCL4L1 stimulation were significantly increased compared to M-CSF stimulation. CD163 is a scavenger receptor required for effective hemoglobin clearance after plaque hemorrhage, and its engagement by hemoglobin-haptoglobin complexes results in upregulation of HMOX1, an enzyme with anti-inflammatory effects and linked to atheroprotection [33]. CXCL4 promotes atherogenesis by suppressing CD163 in macrophages [20]. Although CD163 expression was also downregulated on CXCL4L1-stimulated monocytes compared to resting macrophages, *HMOX1* expression in CXCL4L1-stimulated monocytes was increased compared to resting macrophages, whereas its expression was lower in CXCL4-induced macrophages. The lower expression of *IL-1RN* in CXCL4L1-treated monocytes, compared to CXCL4-treated monocytes, is in line with the more inflammatory phenotype of macrophages generated in the presence of CXCL4L1.

MMPs are major macrophage products with important immunological functions, including extracellular matrix remodeling and modulation of chemokine activity by post-translational processing [19,34–36]. When comparing *MMP* gene expression in CXCL4L1-treated monocytes with that of CXCL4-induced macrophages, we found some MMPs being expressed in CXCL4-stimulated monocytes at significantly higher levels (e.g. *MMP-9*) whereas others showing significantly higher expression in CXCL4L1-differentiated macrophages (e.g. *MMP-2* and *MMP-8*). It is reasonable to assume that the two chemokine variants have differential effects on macrophage-dependent MMP activities, though these results do not allow for a direct estimate of functional effects as the enzymatic activity of MMPs is tightly regulated at several levels, including transcriptional control and post-translational inhibition by tissue inhibitors of metalloproteinases (TIMPs) [37,38].

Dendritic cells are crucial initiators of adaptive immune responses. A range of different inflammatory stimuli can induce monocyte differentiation to dendritic cells, and not surprisingly, iMDDC generated by different inflammatory stimuli may differ in their functions [39]. We here report that CXCL4L1 also influences the differentiation of monocytes into dendritic cells, and specifically the expression of *MMP* genes. As reported previously, CXCL4 significantly inhibited the expression of CD1a on iMDDC [31]. In contrast, the expression of CD1a on iMDDC A differentiated with CXCL4L1 was comparable to GM-CSF/IL-4 stimulated iMDDC. Expression levels of CD80, CD11b, and CD209 were not influenced by CXCL4 or CXCL4L1. Differently from previous reports, this was also observed for CD86 levels [31], a discrepancy possibly due to different time length of CXCL4 exposure.

Different stages of monocyte differentiation are characterized by different repertoires of GPCR. For instance, CCR2 is rapidly downregulated during monocyte differentiation to iMDDC, although the expression of CCR1 and CCR5 is upregulated [28]. LPS and IFN-γ up-regulate CCR7 and downregulate CCR1 in mature dendritic cells [40,41]. Presumably, this reciprocal regulation underlies the trafficking of dendritic cells to lymph nodes through lymphatic vessels to present antigen in the lymph nodes. However, CXCL4 and CXCL4L1 did not affect chemokine receptors expression on iMDDC A. Moreover, the cytokine production profile of CXCL4- or CXCL4L1-differentiated iMDDC A was similar, confirming previously published data describing that CXCL4-treated DC secreted similar amounts of IL-10 compared to control dendritic cells [31]. However, the effect of CXCL4 or CXCL4L1 on the chemokine footprint and/or phagocytic capacity of iMDDCs depended on the time of exposure of iMDDCs to CXCL4 or CXCL4L1.

In the present study, we provide insight into the effect of CXCL4 and CXCL4L1 on monocyte differentiation into macrophages and dendritic cells. We can conclude that differently from CXCL4, CXCL4L1 is not a survival factor for monocytes and that the two chemokine variants also induce distinct macrophage phenotypes different from M1 and M2, with more pronounced inflammatory traits for CXCL4L1. Moreover, iMDDC A developed in the presence of CXCL4 showed a phenotype different from that seen in CXCL4L1 differentiated iMDDC A, i.e. enhanced phagocytic capacity and downregulation of expression of certain surface markers (e.g. CD1a) and enzymes (e.g. *MMP-9* and *MMP-12*). In contrast, in CXCL4-induced macrophages *MMP-9* expression was upregulated. Taken together, these results candidate CXCL4 and CXCL4L1 as distinct platelet-derived mediators during inflammatory reactions. However, it is hard to explore the *in vivo* consequences of these *in vitro* observations, as the two *CXCL4* and *CXCL4L1* genes are only present in primates and mice only have one *CXCL4* gene. The murine CXCL4 might combine properties of human CXCL4 and CXCL4L1.

Nevertheless, it has been previously suggested that modulation of macrophage heterogeneity by CXCL4 may represent an interesting therapeutic approach [42–44]. In atherosclerosis for example, driving macrophages towards an atheroprotective phenotype by treatments targeting platelet-derived chemokines may help to limit disease progression and improve long-term prognosis in patients with cardiovascular diseases [42–44]. Furthermore, it is generally accepted that macrophages are crucial stromal cells influencing tumor development [7–10]. In view of the effect of CXCL4 and CXCL4L1 on macrophages and dendritic cells, the tumor microenvironment can also be shaped by those platelet chemokines. We observed selective CXCL4L1 expression in colon cancer patients by immunohistochemistry in tumor cells [45] but the number of specimens analyzed was too low to allow correlation analyses with tumor stage or survival. Furuya *et al*. detected high levels of CXCL4 and CXCL4L1 mRNA in endometriosis, an inflammatory status that can evolve to ovarian cancer [46]. Interestingly, CXCL4 (and/or CXCL4L1, because a non-discriminating antibody was used) expression decreased in tumor-associated macrophages (TAMs) of ovarian cancers arising in endometriosis [46]. Similarly, CXCL4 production decreased in lung Ly6G^+^CD11b^+^ cells in a murine model of breast cancer [47]. Concomitant with evolution from normal lungs towards lungs carrying breast cancer or melanoma metastases, murine CXCL4 diminished at the mRNA and protein level in Ly6G^+^CD11b^+^ cells. In breast cancer patients, global tumor CXCL4 mRNA expression levels were negatively correlated with tumor stage and positively correlated with patient survival [47]. In conclusion, the multipotent nature of CXCL4 and CXCL4L1 implies that those platelet chemokines affect, apart from angiogenesis, also lymphocyte recruitment and monocyte/macrophage survival or polarization in the tumor stroma.

## References

[1] Parihar A, Eubank TD, Doseff AI. Monocytes and macrophages regulate immunity through dynamic networks of survival and cell death. J Innate Immun. 2010;2: 204–215. 10.1159/000296507 20375558 PMC2956013

[2] Gordon S. Targeting a monocyte subset to reduce inflammation. Circ Res. 2012;110: 1546–1548. 10.1161/RES.0b013e31825ec26d 22679136

[3] Martinez FO, Gordon S, Locati M, Mantovani A. Transcriptional profiling of the human monocyte-to-macrophage differentiation and polarization: new molecules and patterns of gene expression. J Immunol. 2006;177: 7303–7311. 17082649 10.4049/jimmunol.177.10.7303

[4] Bellora F, Castriconi R, Doni A, Cantoni C, Moretta L, Mantovani A, et al M-CSF induces the expression of a membrane-bound form of IL-18 in a subset of human monocytes differentiating *in vitro* toward macrophages. Eur J Immunol. 2012;42: 1618–1626. 10.1002/eji.201142173 22678914

[5] Murray PJ, Allen JE, Biswas SK, Fisher EA, Gilroy DW, Goerdt S, et al Macrophage activation and polarization: nomenclature and experimental guidelines. Immunity. 2014;41: 14–20. 10.1016/j.immuni.2014.06.008 25035950 PMC4123412

[6] Mantovani A, Sica A, Sozzani S, Allavena P, Vecchi A and Locati M. The chemokine system in diverse forms of macrophage activation and polarization. Trends Immunol. 2004;25: 677–686. 10.1016/j.it.2004.09.015 15530839

[7] Mantovani A, Sozzani S, Locati M, Allavena P, Sica A. Macrophage polarization: tumor-associated macrophages as a paradigm for polarized M2 mononuclear phagocytes. Trends Immunol. 2002;23: 549–555. 12401408 10.1016/s1471-4906(02)02302-5

[8] Varin A and Gordon S. Alternative activation of macrophages: immune function and cellular biology. Immunobiology. 2009;214: 630–641. 10.1016/j.imbio.2008.11.009 19264378

[9] Biswas SK, Sica A, Lewis CE. Platicity of macrophage function during tumor progression: regulation by distinct molecular mechanisms. J Immunol. 2008;180: 2011–2017. 18250403 10.4049/jimmunol.180.4.2011

[10] Murray PJ and Wynn TA. Protective and pathogenic functions of macrophage subsets. Nat Rev Immunol. 2011;11: 723–737. 10.1038/nri3073 21997792 PMC3422549

[11] Kameyoshi Y, Dörschner A, Mallet AI, Christophers E, Schröder JM. Cytokine RANTES released by thrombin-stimulated platelets is a potent attractant for human eosinophils. J Exp Med. 1992;176: 587–592. 1380064 10.1084/jem.176.2.587 PMC2119329

[12] Gleissner CA, von Hundelshausen P, Ley K. Platelet chemokines in vascular disease. Arterioscler Thromb Vasc Biol. 2008; 28: 1920–1927. 10.1161/ATVBAHA.108.169417 18723831 PMC2657037

[13] Kasper B and Petersen F. Molecular pathways of platelet factor 4/CXCL4 signaling. Eur J Cell Biol. 2011;90: 521–526. 10.1016/j.ejcb.2010.12.002 21295372

[14] Nesmelova IV, Sham Y, Gao J, Mayo KH. CXC and CC chemokines form mixed heterodimers: association free energies from molecular dynamics simulations and experimental correlations. J Biol Chem. 2008;283: 24155–24166. 10.1074/jbc.M803308200 18550532 PMC2527121

[15] Lasagni L, Francalanci M, Annunziato F, Lazzeri E, Giannini S, Cosmi L, et al An alternatively spliced variant of CXCR3 mediates the inhibition of endothelial cell growth induced by IP-10, Mig, and I-TAC, and acts as functional receptor for platelet factor 4. J Exp Med. 2003;197: 1537–1549. 10.1084/jem.20021897 12782716 PMC2193908

[16] Mueller A, Meiser A, McDonagh EM, Fox JM, Petit SJ, Xanthou G, et al CXCL4-induced migration of activated T lymphocytes is mediated by the chemokine receptor CXCR3. J Leukoc Biol. 2008; 83: 875–882. 10.1189/jlb.1006645 18174362

[17] Van Raemdonck K, Gouwy M, Lepers SA, Van Damme J, Struyf S. CXCL4L1 and CXCL4 signaling in human lymphatic and microvascular endothelial cells and activated lymphocytes: involvement of mitogen-activated protein (MAP) kinases, Src and p70S6 kinase. Angiogenesis. 2014;17: 631–640. 10.1007/s10456-014-9417-6 24469069

[18] Scheuerer B, Ernst M, Dürrbaum-Landmann I, Fleischer J, Grage-Griebenow E, Brandt E, et al The CXC-chemokine platelet factor 4 promotes monocyte survival and induces monocyte differentiation into macrophages. Blood. 2000;95: 1158–1166. 10666185

[19] Gleissner CA, Shaked I, Little KM, Ley K. CXCL4 induces a unique transcriptome in monocyte-derived macrophages. J Immunol. 2010;184: 4810–4818. 10.4049/jimmunol.0901368 20335529 PMC3418140

[20] Gleissner CA, Shaked I, Erbel C, Bockler D, Katus HA, Ley K. CXCL4 downregulates the atheroprotective hemoglobin receptor CD163 in human macrophages. Circ Res. 2010;106: 203–211. 10.1161/CIRCRESAHA.109.199505 19910578 PMC2876722

[21] Fricke I, Mitchell D, Petersen F, Böhle A, Bulfone-Paus S, Brandau S. Platelet factor 4 in conjunction with IL-4 directs differentiation of human monocytes into specialized antigen-presenting cells. FASEB J. 2004;18: 1588–1590. 10.1096/fj.03-1435fje 15319366

[22] Luster AD, Greenberg SM, Leder P. The IP-10 chemokine binds to a specific cell surface heparin sulfate site shared with platelet factor 4 and inhibits endothelial cell proliferation. J Exp Med. 1995;182: 219–231. 7790818 10.1084/jem.182.1.219 PMC2192102

[23] Tanaka T, Manome Y, Wen P, Kufe DW, Fine HA. Viral vector-mediated transduction of a modified platelet factor 4 cDNA inhibits angiogenesis and tumor growth. Nat Med. 1997;3: 437–442. 9095178 10.1038/nm0497-437

[24] Strieter RM, Burdick MD, Mestas J, Gomperts B, Keane MP, Belperio JA. Cancer CXC chemokine networks and tumour angiogenesis. Eur J Cancer. 2006;42: 768–778. 10.1016/j.ejca.2006.01.006 16510280

[25] Vandercappellen J, Van Damme J, Struyf S. The role of the CXC chemokines platelet factor-4 (CXCL4/PF-4) and its variant (CXCL4L1/PF-4var) in inflammation, angiogenesis and cancer. Cytokine Growth Factor Rev. 2011;22: 1–18. 10.1016/j.cytogfr.2010.10.011 21111666

[26] Struyf S, Burdick MD, Proost P, Van Damme J, Strieter RM. Platelets release CXCL4L1, a nonallelic variant of the chemokine platelet factor-4/CXCL4 and potent inhibitor of angiogenesis. Circ Res. 2004;95: 855–857. 10.1161/01.RES.0000146674.38319.07 15459074

[27] Struyf S, Burdick MD, Peeters E, Van den Broeck K, Dillen C, Proost P, et al Platelet factor-4 variant chemokine CXCL4L1 inhibits melanoma and lung carcinoma growth and metastasis by preventing angiogenesis. Cancer Res. 2007;67: 5940–5948. 10.1158/0008-5472.CAN-06-4682 17575164

[28] Gouwy M, Struyf S, Leutenez L, Pörtner N, Sozzani S, Van Damme J. Chemokines and other GPCR ligands synergize in receptor-mediated migration of monocyte-derived immature and mature dendritic cells. Immunobiology. 2014;219: 218–229. 10.1016/j.imbio.2013.10.004 24268109

[29] Livak KJ, Schmittgen TD. Analysis of relative gene expression data using real-time quantitative PCR and the 2(-Delta Delta C(T)) method. Methods. 2001;25: 402–408. 10.1006/meth.2001.1262 11846609

[30] Schutyser E, Struyf S, Proost P, Opdenakker G, Laureys G, Verhasselt B, et al Identification of biologically active chemokine isoforms from ascitic fluid and elevated levels of CCL18/pulmonary and activation-regulated chemokine in ovarian carcinoma. J Biol Chem. 2002;277: 24584–24593. 10.1074/jbc.M112275200 11978786

[31] Xia CQ, Kao KJ. Effect of CXC chemokine platelet factor 4 on differentiation and function of monocyte-derived dendritic cells. Int Immunol. 2003;15: 1007–1115. 12882838 10.1093/intimm/dxg100

[32] Schwartzkopff F, Petersen F, Grimm TA, Brandt E. CXC chemokine ligand 4 (CXCL4) down-regulates CC chemokine receptor expression on human monocytes. Innate Immun. 2012;18: 124–139. 10.1177/1753425910388833 21088050

[33] Schaer CA, Schoedon G, Imhof A, Kurrer MO, Schaer DJ. Constitutive endocytosis of CD163 mediates hemoglobin-heme uptake and determines the noninflammatory and protective transcriptional response of macrophages to hemoglobin. Circ Res. 2006;99: 943–950. 10.1161/01.RES.0000247067.34173.1b 17008602

[34] Goetzl EJ, Banda MJ, Leppert D. Matrix metalloproteinases in immunity. J Immunol. 1996;156: 1–4. 8598448

[35] Mortier A, Van Damme J, Proost P. Regulation of chemokine activity by posttranslational modification. Pharmacol Ther. 2008;120: 197–217. 10.1016/j.pharmthera.2008.08.006 18793669

[36] Erbel C, Tyka M, Helmes CM, Akhavanpoor M, Rupp G, Domschke G, et al CXCL4-induced plaque macrophages can be specifically identified by co-expression of MMP7+S100A8+ in vitro and in vivo. Innate Immun. 2015;21: 255–265. 10.1177/1753425914526461 24663337

[37] Newby AC. Metalloproteinase expression in monocytes and macrophages and its relationship to atherosclerotic plaque instability. Arterioscler Thromb Vasc Biol. 2008;28: 2108–2114. 10.1161/ATVBAHA.108.173898 18772495

[38] Vandooren J, Born B, Solomonov I, Zajac E, Saldova R, Senske M, et al Circular trimers of gelatinase B/matrix metalloproteinase-9 constitute a distinct population of functional enzyme molecules differentially regulated by tissue inhibitor of metalloproteinases-1. Biochem J. 2015;465: 259–270. 10.1042/BJ20140418 25360794 PMC4399976

[39] Auffrey C, Sieweke MH, Geissmann F. Blood monocytes: development, heterogeneity and relationship with dendritic cells. Annu Rev Immunol. 2009;27: 669–692. 10.1146/annurev.immunol.021908.132557 19132917

[40] Sallusto F, Schaerli P, Loetscher P, Schaniel C, Lenig D, Mackay CR, et al Rapid and coordinated switch in chemokine receptor expression during dendritic cell maturation. Eur J Immunol. 1998;28: 2760–2769. 10.1002/(SICI)1521-4141(199809)28:09<2760::AID-IMMU2760>3.0.CO;2-N 9754563

[41] Sozzani S, Allavena P, D'Amico G, Luini W, Bianchi G, Kataura M, et al Differential regulation of chemokine receptors during dendritic cell maturation: a model for their trafficking properties. J Immunol. 1998;161: 1083–1086. 9686565

[42] Colin S, Chinetti-Gbaguidi G, Staels B. Macrophage phenotypes in atherosclerosis. Immunol Rev. 2014;262: 153–166. 10.1111/imr.12218 25319333

[43] Gleissner CA. Platelet-derived chemokines in atherogenesis: what’s new? Curr Vasc Pharmacol. 2012;10: 563–569. 22338571 10.2174/157016112801784521

[44] Gleissner CA. Macrophage Phenotype Modulation by CXCL4 in atherosclerosis. Front Physiol. 2012;3: 1–7. 10.3389/fphys.2012.00001 22275902 PMC3257836

[45] Verbeke H, De Hertogh G, Li S, Vandercappellen J, Noppen S, Schutyser E, et al Expression of angiostatic platelet factor-4var/CXCL4L1 counterbalances angiogenic impulses of vascular endothelial growth factor, interleukin-8/CXCL8, and stromal cell-derived factor 1/CXCL12 in esophageal and colorectal cancer. Hum Pathol. 2010;41: 990–1001. 10.1016/j.humpath.2009.09.021 20334899

[46] Furuya M, Tanaka R, Miyagi E, Kami D, Nagahama K, Miyagi Y, et al Impaired CXCL4 expression in tumor-associated macrophages (TAMs) of ovarian cancers arising in endometriosis. Cancer Biol Ther. 2012;13: 671–680. 10.4161/cbt.20084 22555803 PMC3408972

[47] Jian J, Pang Y, Yan HH, Min Y, Achyut BR, Hollander MC, et al Platelet factor 4 is produced by subsets of myeloid cells in premetastatic lung and inhibits tumor metastasis. Oncotarget 2016; in press. 10.18632/oncotarget.9486 PMC5438604 27223426

